# Epidemiology of intussusception in infants less than one year of age in Ghana, 2012-2016

**DOI:** 10.11604/pamj.supp.2021.39.1.25445

**Published:** 2021-07-29

**Authors:** Hope Glover-Addy, Daniel Ansong, Christabel Enweronu-Laryea, Jacqueline E. Tate, Kwame Amponsa-Achiano, Badu Sarkodie, Jason M Mwenda, Stanley Diamenu, Sandra Kwarteng Owusu, Boateng Nimako, Nicholas Karikari Mensah, Joseph Armachie, Clement Narh, Kimberly Pringle, Scott P Grytdal, Fred Binka, Ben Lopman, Umesh D Parashar, George Armah

**Affiliations:** 1 Department of Surgery, Korle Bu Teaching Hospital, Accra, Ghana,; 2 Department of Child Health, Komfo Anokye Teaching Hospital and School of Medicine Dentistry, Kumasi, Ghana,; 3 Department of Child Health, Korle Bu Teaching Hospital, Accra, Ghana,; 4 CDC, Atlanta, USA,; 5 Ministry of Health, Ghana,; 6 World Health Organization,; 7 Noguchi Medical Institute for Medical Research, University of Ghana, Accra, Ghana,; 8 University of Allied Health Sciences, Ho, Ghana,; 9 Department of Global Health, Emory University, Atlanta, USA

**Keywords:** Epidemiology, intussusception, Ghana

## Abstract

**Introduction:**

we examined the epidemiology, clinical and demographic characteristics of intussusception in Ghanaian infants.

**Methods:**

active sentinel surveillance for pediatric intussusception was conducted at Komfo Anokye Teaching Hospital in Kumasi and Korle Bu Teaching Hospital in Accra. From March 2012 to December 2016, infants < 1 year of age who met the Brighton Collaboration level 1 diagnostic criteria for intussusception were enrolled. Data were collected through parental interviews and medical records abstraction.

**Results:**

a total of 378 children < 1 year of age were enrolled. Median age at onset of intussusception was 27 weeks; only 12 cases (1%) occurred in infants < 12 weeks while most occurred in infants aged 22-34 weeks. Median time from symptom onset until referral to a tertiary hospital was 2 days (IQR: 1-4 days). Overall, 35% of infants were treated by enema, 33% had surgical reduction and 32% required surgical reduction and bowel resection. Median length of hospital stay was 5 days (IQR: 3-8 days) with most patients (95%) discharged home. Eleven (3%) infants died. Infants undergoing enema reduction were more likely than those treated surgically to present for treatment sooner after symptom onset (median 1 vs 3 days; p < 0.0001) and have shorter hospital stays (median 3 vs 7 days; p < 0.001).

**Conclusion:**

Ghanaian infants had a relatively low case fatality rate due to intussusception, with a substantial proportion of cases treated non-surgically. Early presentation for treatment, possibly enhanced by community-based health education programs and health information from various media platforms during the study period might contribute to both the low fatality rate and high number of successful non-surgical treatments in this population.

## Introduction

Intussusception, a condition in which a segment of the intestine invaginates into the adjourning intestinal lumen, is the most common cause of bowel obstruction in young children. The global incidence of intussusception in infants is 74 per 100,000 and varies considerably by region.1 In Africa, few incidence estimates are available; one study estimated a rate of 56 per 100,000 infants < 1 year of age in South Africa [1, 2]. The overall case fatality rate for intussusception cases is 9% in the African region but rates have ranged as high as 28-34% in some countries [1, 3, 4]. The cause of most intussusception cases is not known but some infectious agents, particularly respiratory adenoviruses, have been associated with intussusception [5, 7]. If treatment is sought early, intussusception can be reduced by enema. If treatment is sought late or if facilities with enema reduction capabilities are not available, surgical reduction is required. Intussusception can be fatal if untreated.

In some high- and middle-income settings rotavirus vaccines have been associated with a small, increased risk of intussusception after the first dose of vaccine [8-14]. Subsequently, the African Intussusception Surveillance Network was established in 2012 in 7 sub-Saharan African countries including Ghana to examine the risk of intussusception following monovalent rotavirus vaccination in this region [15]. No increased risk of intussusception following rotavirus vaccination was observed in a pooled analysis of data from these 7 countries or in a similar analysis in South Africa [15, 16]. However, descriptions of the epidemiology of intussusception in sub-Saharan Africa remain limited. This paper seeks to describe the clinical and demographic characteristics of the intussusception cases enrolled in Ghana as part of the African Intussusception Surveillance Network.

## Methods

Active sentinel surveillance for intussusception was conducted at two large tertiary care hospitals in Ghana. Komfo Anokye Teaching Hospital (KATH), Kumasi, is a 1200 bed hospital in the middle belt of the country; it has 25 pediatric surgical beds and enrolled patients from March 2012 to December 2016. Korle Bu Teaching Hospital (KBTH), Accra, is a 2000 bed facility in the southern belt; the40-bedspediatric surgical unit enrolled patients from September 2013 to December 2016.

Infants < 1 years of age meeting the Brighton Collaboration criteria for level 1 of diagnostic certainty for intussusception [17] were enrolled. To meet Level 1 of diagnostic certainty, the intussusception must be confirmed during surgery, by specific radiologic findings (if reduced by enema), or at autopsy. A case report form collecting limited demographic and treatment information was completed for each case by review of the infant´s medical record and through interview of the infant´s parents or guardians. Descriptive statistics including frequencies and medians were used to summarize the data. Wilcoxon rank-sum tests were used to compare for continuous variables and χ2 tests to compare categorical variables. A p-value of < 0.05 was considered statistically significant.

**Disclaimer:** the findings and conclusions in this report are those of the authors and do not necessarily represent the official position of the Centers for Disease Control and Prevention (CDC) or the World Health Organization.

## Results

**Figure 1:**
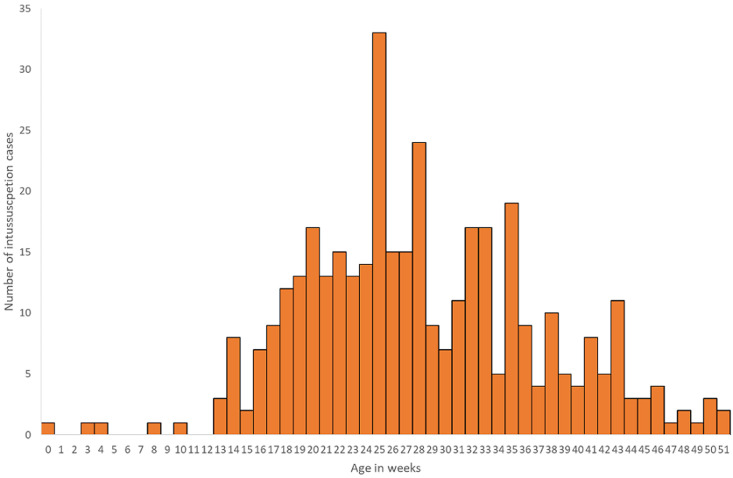
age distribution of infants diagnosed with intussusception, Korle Bu (KBTH) and Komfo Anokye (KATH) Teaching Hospitals, Ghana, 2012-2016

**Figure 2:**
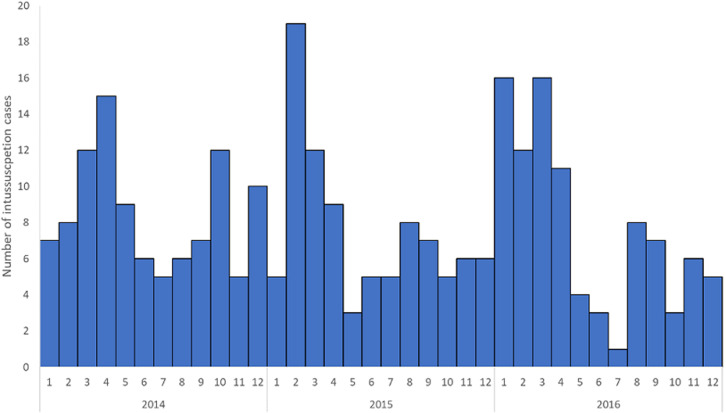
seasonality by month and year of intussusception cases, Korle Bu (KBTH) and Komfo Anokye (KATH) Teaching Hospitals, Ghana, 2012-2016

A total of 378 intussusception cases in children < 1 year of age that met the Brighton Collaboration criteria for level 1 of diagnostic certainty for intussusceptions were enrolled from the two surveillance sites. KATH enrolled 155 (41%) infants and KBTH enrolled 223 (59%) infants (Table 1). Just over half (53%) of infants were male. The median age at intussusception onset was 27 weeks. Intussusception was rare in young infants with only 12 cases (1%) occurring in infants < 12 weeks of age. Intussusception cases peaked in infants 22 to 34 weeks of age before gradually declining in older infants (Figure 1). Cases occurred throughout the year with an increase in the number of cases enrolled during January to April of each year (Figure 2).

For most (94%) patients, clinicians recognized clinical symptoms of intussusception when making their diagnosis (Table 2). More than two-thirds of cases (68%) had an ultrasound preformed. At least 130 infants were transferred from another facility to one of the two surveillance hospitals for treatment with a median of 1 day (interquartile range (IQR): 0-2 days) between admission at the first facility and the surveillance facility. The median time from symptom onset until admission at the surveillance facility was 2 days (IQR: 1-4 days) with 72 (19%) infants presenting on the same day as symptom onset and 80 (21%) presenting on the following day. Just over one third (35%) of the infants were treated by enema and the remaining infants were treated surgically. Of those treated surgically, approximately half (49%) required resection of the bowel. The median length of hospital stay was 5 days (IQR: 3-8 days) with most (95%) patients discharged home. Eleven (3%) intussusception patients died. No differences in age or sex were observed between infants undergoing enema reduction compared with infants undergoing surgical reduction (Table 3). Infants undergoing enema reduction were more likely to present to the surveillance facility for treatment sooner after symptom onset (median 1 day (IQR: 0-3)) than infants undergoing surgical reduction (median 3 days (IQR: 1-4); p < 0.0001). Similarly, infants with enema reduction had shorter hospital stays (median 3 days (IQR: 2-4)) than infants with surgical reduction (median 7 days (IQR: 5-10); p < 0.001). No infants with enema reduction died; versus 8 (4%) infants with surgical reduction.

**Table 1:** demographic characteristics of children with intussusception at the Korle Bu (KBTH) and Komfo Anokye (KATH) Teaching Hospitals in Ghana, 2012-2016

	n/N (%)
**Enrollment Site**	
KATH	155/378 (41%)
KBTH	223/378 (59%)
**Male**	200/378 (53%)
**Median age (in weeks) at intussusception onset** (IQR)	27 (22-34)
**Age (in weeks) at intussusception onset**	
0-11 weeks	12/378 (1%)
12-23 weeks	112/378 (30%)
24-35 weeks	168/378 (49%)
36+ weeks	75/378 (20%)
**Ever Breastfed**	322/324 (99%)
**Median age (in months) for initiation of milk (other than breastmilk)†**(IQR)	4 (3-6)
**Median age (in months) for initiation of solid food‡**(IQR)	6 (4-6)
Median birthweight in (kilograms)* (IQR)	3.2 (2.9-3.5)

† Among 150 children who had initiated milk other than breastmilk; 14 (4%) children had not initiated other milk; information missing for 214 (57%) of infants ‡Among 114 children who had initiated solid foods; 45 (12%) children had not initiated solid foods; information missing for 219 (58%) of infants *Among 265 infants with non-missing birthweight

**Table 2:** clinical characteristics of children with intussusception at the Korle Bu (KBTH) and Komfo Anokye (KATH) Teaching Hospitals in Ghana, 2012-2016

	n/N (%)
**Diagnosis of Intussusception‡**	
Clinical symptoms	355/378 (94%)
Enema	23/378 (6%)
Ultrasound	256/378 (68%)
Surgery	149/378 (39%)
Median number of days (IQR) between admission to first facility and admission to surveillance facility*	1 (0-2)
**Median number of days (IQR) between symptom onset and admission to surveillance facility**	2 (1-4)
**Treatment of Intussusception**	
Enema	132/374 (35%)
Surgery	242/374 (65%)
**Among children with surgery, n (%) that required resection**	116/237 (49%)
**Median length of hospital stay in days† (IQR)**	5 (3-8)
**Outcome**	
Discharged home	331/348 (95%)
Transferred	6/348 (2%)
Died	11/348 (3%)

**‡**Diagnosis categories are not mutually exclusive and multiple pieces of information could be used to make a diagnosis *Among 126 infants with information on the date of admission to the first facility and to the surveillance facility †Among 356 infants with non-missing admission and discharge dates

**Table 3:** comparison of intussusception cases undergoing enema reduction versus surgical reduction, Korle Bu (KBTH) and Komfo Anokye (KATH) Teaching Hospitals, Ghana, 2012-2016

	Enema Reduction (n=132)	Surgical Reduction (n=242)	p-value
**Male**	69 (52%)	128 (53%)	0.91
**Enrollment Site**			0.06
KATH	45 (34%)	107 (44%)	
KBTH	87 (66%)	135 (55%)	
**Median age (in weeks) at intussusception onset** (IQR)	28 (23-37)	27 (22-33)	0.10
**Median number of days (IQR) between symptom onset and admission to surveillance facility**	1 (0-3)	3 (1-4)	<0.0001
**Median length of hospital stay in days (IQR)**	3 (2-4)	7 (5-10)	<0.0001
**Outcome**			0.11
Discharged home	119 (98%)	211 (95%)	
Transferred	2 (2%)	4 (2%)	
Died	0 (0%)	8 (4%)	

## Discussion

More than one third of intussusception cases were reduced non-surgically at these two large tertiary care hospitals in Ghana. The enema-reduced intussusception cases were more likely to have presented to the surveillance facility for treatment sooner and had short hospital stays than cases that were treated surgically. None of the enema-reduced intussusception cases died. These findings highlight the importance of prompt access to care and availability of non-surgical methods for reduction of intussusception in reducing the morbidity and mortality due to this condition. Intussusception cases that presented late tended to require surgical reduction and had poorer outcomes. Compared with other countries in sub-Saharan Africa where case fatality rates for intussusception often exceed 10% and can be as high as 25%-33%, Ghana´s intussusception case fatality rate of 3% in this evaluation is starkly lower and reflects this access to care and non-surgical reduction methods [1, 3, 4, 18-20]. Additionally, there was a concerted effort to educate health workers and the public about intussusception following the introduction of rotavirus vaccine. The early referral to tertiary centers may also be a result of this drive to improve health literacy through community-based health education programs and health information from various media platforms. Overall, intussusception cases in Ghana presented for care 2 days after symptom onset compared with > 3 days as has been observed in many African countries [21].

Another key finding is that intussusception cases were rare in the first 3 months of life with only 1% of cases occurring before 12 weeks of age. The age distribution of intussusception cases in Ghana is similar to that from a previous analysis in Ghana as in other countries with relatively few cases in early infancy followed by a peak around 6 months of age before tapering downward in older infancy [1, 22]. Rotavirus vaccine is recommended in Ghana at 6 and 10 weeks of age and on-time vaccination will ensure that the vaccine is administered at a time when the risk of intussusception is naturally low.

This analysis has several limitations. First, this was a secondary analysis of data collected to answer a specific question regarding the association between rotavirus vaccination and intussusception. No association between vaccination and intussusception was observed after either dose of vaccine in the larger analysis pooled across multiple countries [15]. Only a limited number of variables were collected for each case which restricts the clinical and epidemiological description of these cases. However, we were able to identify a few unique, informative factors related to the treatment of intussusception in Ghana. Second, surveillance for intussusception was only conducted at two facilities in Ghana. Both facilities were large teaching hospitals and may not be representative of the care received by all intussusception cases in Ghana. However, they do exemplify the level of care that can be provided with sufficient training and facilities.

## Conclusion

In summary, observed case fatality rates from intussusception in Ghana were low compared with other countries in Africa. Prompt presentation and diagnosis of intussusception enable cases of intussusception to be treated non-surgically resulting in shorter hospital stays and thereby reducing the burden on the healthcare system.

### What is known about this topic

- Intussusception is the most common cause of bowel obstruction in young children;
- The cause of most intussusception is not known but some infectious agents, particularly respiratory adenoviruses, have been associated with intussusception;
- The global incidence of intussusception in infants varies considerably by region; however, descriptions of the epidemiology of intussusception in sub-Saharan Africa remain very limited.

### What this study adds

- Intussusception cases in Ghana rare in the first 3 months of life;
- Case fatality rates from intussusception in Ghana were low compared with other countries in Africa;
- Early presentation for treatment, possibly enhanced by community-based health education programs and health information might have contributed to both the low fatality rate and high number of successful non-surgical treatments reported in this study.
